# Neural Activation and Functional Connectivity during Motor Imagery of Bimanual Everyday Actions

**DOI:** 10.1371/journal.pone.0038506

**Published:** 2012-06-06

**Authors:** André J. Szameitat, Adam McNamara, Shan Shen, Annette Sterr

**Affiliations:** 1 Department of Psychology, Ludwig Maximilians University, Munich, Germany; 2 Department of Psychology, University of Surrey, Stag Hill, Guildford, United Kingdom; University of Bologna, Italy

## Abstract

Bimanual actions impose intermanual coordination demands not present during unimanual actions. We investigated the functional neuroanatomical correlates of these coordination demands in motor imagery (MI) of everyday actions using functional magnetic resonance imaging (fMRI). For this, 17 participants imagined unimanual actions with the left and right hand as well as bimanual actions while undergoing fMRI. A univariate fMRI analysis showed no reliable cortical activations specific to bimanual MI, indicating that intermanual coordination demands in MI are not associated with increased neural processing. A functional connectivity analysis based on psychophysiological interactions (PPI), however, revealed marked increases in connectivity between parietal and premotor areas within and between hemispheres. We conclude that in MI of everyday actions intermanual coordination demands are primarily met by changes in connectivity between areas and only moderately, if at all, by changes in the amount of neural activity. These results are the first characterization of the neuroanatomical correlates of bimanual coordination demands in MI. Our findings support the assumed equivalence of overt and imagined actions and highlight the differences between uni- and bimanual actions. The findings extent our understanding of the motor system and may aid the development of clinical neurorehabilitation approaches based on mental practice.

## Introduction

Motor imagery (MI) refers to the mental rehearsal of a movement without overtly performing the respective action [1]. It provides an intriguing way to learn and improve motor acts and as such has a number of applications in neurorehabilition, sports, and artistic performance. Moreover, MI is an excellent tool to study the functionality of the motor system beyond simple motor acts easily performed in laboratory settings. Consequently, a vast amount of research has been conducted characterizing MI. One basic pattern of results is that MI and overt motor execution (ME) draw on similar cognitive and neural mechanisms, which is in line with theoretical accounts of MI [2], [3]. This notion of equivalence is well evidenced for a range of parameters such as speed-accuracy tradeoff [4], corticomotor excitability [5], [6], cortical surface activity [7], and advanced motor preparation [8], as well as the network of brain areas controlling motor functions [3], [9]–[11]. However, some characteristics of MI remain largely unexplored.

In particular, in our everyday life many actions are bimanual in nature, such as tying shoelaces, folding a sheet of paper, or buttoning a shirt. Such actions require that both hands move cooperatively. For instance, when tying shoelaces the hands interact so closely that the movement of one hand is meaningless without the accompanying movements of the other hand. This strong coupling and inter-dependence requires additional processes related to the coordination of both limbs, which are not required during unimanual actions [12], [13]. Since these demands in bimanual actions exceed what would be expected by the mere sum of two separate unimanual actions, we consider them as “over-additive”. The bimanual coordination processes may be realized by at least two (non-exclusive) mechanisms. First, they may be realized by increased neural activity, resulting in increased BOLD signal as measured by fMRI. Second, they may be realized by a change in how brain areas are functionally connected with each other, resulting in changed functional connectivity as measured by psychophysiological interactions. The aim of the present study was to identify these additional demands in MI of everyday tasks and to test by which mechanism(s) they are realized.

Since no previous study investigated this particular question, hypotheses can only be derived from related research. For instance, Grefkes et al. [14] has shown that overt bimanual movements result in activation of the SMA and increased connectivity between areas of the motor system as assessed by structural equation modeling (SEM). However, the activity of the SMA actually did not seem to have exceeded the activity expected by the mere sum of left and right hand actions and therefore may not be related to bimanual coordination at all [14], [15]. This is in line with Puttemans et al. [16] who showed that overt performance of overlearned bimanual movements induced activations related to bimanual coordination effort only in two sub-cortical but no cortical areas. Consequently, we predicted that the bimanual coordination demands in MI are reflected only to a small extent, if at all, by changes in cortical activation, and that they are predominantly reflected by changes in functional connectivity.

We chose to use MI of everyday tasks instead of more simplistic laboratory actions for a number of reasons [17]. First, theoretical accounts of MI strongly depend on the equivalence of MI and overt execution [2], [3]. Therefore, it is important from a theoretical point of view to confirm that also MI of ecologically valid everyday tasks show characteristics found in overt performance. Second, MI is widely used in applied fields such as motor rehabilitation and sports, for instance in the form of mental practice. However, while these applications often use complex everyday tasks their theoretical foundation is based on highly simplistic laboratory tasks such as fist making or button presses, which may be an invalid transfer [18].

Presently, it is an open question in how far the results gained by rather simplistic laboratory tasks (e.g. fist making [14]) can be generalized to ecologically valid everyday tasks, because the demands on bimanual coordination differ profoundly. When tying shoelaces, as mentioned above, the hands need to be tightly coordinated to form a coherent meaningful action. When participants are instructed to simultaneously make fists, at most the movement onsets need to be coordinated, while there is no further demand for continuous bimanual coordination. On the other hand, an often employed simple modification to the task instruction can make fist making a highly demanding task, that is asking the participants to perform the cyclic movement of each hand with a different frequency (e.g. make a fist three times with the left hand while only two times with the right hand, a 3∶2 frequency ratio). These tasks, however, seem much more complex and arbitrary than ecologically valid everyday actions, so that their coordination demands may be associated with different neural correlates as compared to everyday actions. Of course all these laboratory tasks have been proven to be highly useful to illuminate the workings of the motor system in bimanual coordination, our point is merely that the results of these previously used tasks may not be generalized to ecologically more valid everyday tasks in a straightforward way.

Here we report data from 17 participants. The analysis is divided into two parts. First, a univariate fMRI analysis is employed to test for condition-specific differences in neural activation [19] and to determine the seed regions for a connectivity analysis. Due to the lack of prior evidence and ambiguity as to whether the results of simple laboratory tasks can be generalized to everyday tasks, we used, in the second part of the analysis, a multi psychophysiologic interaction (mPPI) approach [20]. MPPI is a data-driven approach in that it does not require one to specify *a priori* hypotheses about connectivity profiles (as required e.g. by SEM and by dynamic causal modeling, DCM).

In more detail, participants completed five different kinesthetic motor imagery conditions, MI of bimanual actions (Biman, e.g. tying shoelaces), MI of simple unimanual tasks (e.g. pressing a button) with the left (Simple-L) and right (Simple-R) hand, MI of complex unimanual tasks (e.g. writing) with the left (Complex-L) and right (Complex-R) hand, and a resting baseline (Baseline). We included two types of unimanual conditions, i.e. simple and complex tasks, because unimanual and bimanual actions may differ in their complexity. In the following the combination of the unimanual conditions Simple-L and Complex-L is referred to as Uni-L, the combination of Simple-R and Complex-R is referred to as Uni-R, and all four unimanual tasks are referred to as Uniman.

## Results

### Behavioral Data

**Figure 1:**
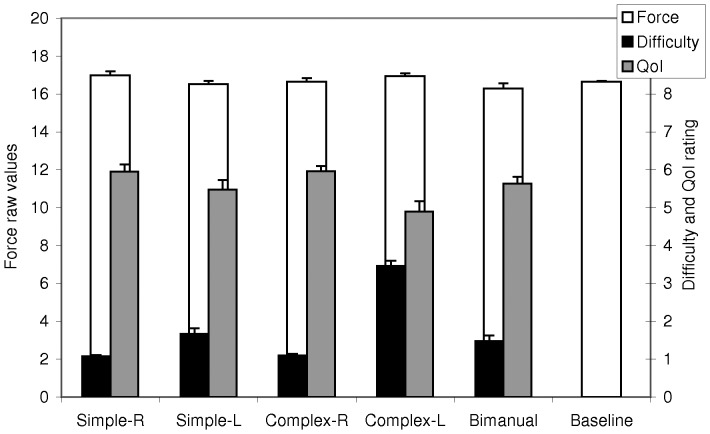
Behavioral data. White bars and left axis denote raw force values averaged across both hands. Gray bars and right axis denote quality of imagination (QoI) rating (rating scale ranged from 1–7). Black bars and right axis denote estimated difficulty of overt performance of the actions (rating scale ranged from 1–5). Note that Difficulty values for the Bimanual condition were derived from an independent sample (see Methods). For illustration purposes interval scale level was assumed for the Difficulty and QoI rating values and means and SEMs are displayed. Error bars denote SEM.

Before the experiment started participants were asked to rate how difficult it would be to overtly perform perform the movements used in the present experiment. Participants rated the perceived difficulty for each movement when performed with the left and right hand, respectively, using a scale ranging from 1 (“very easy”) to 5 (“very hard”). Ratings of different unimanual tasks were significantly different for complex and simple actions (non-parametric Wilcoxon tests (N = 17), see Fig. 1, black bars). Imagination of left hand actions was perceived to be more difficult than right hand actions for both categories of complexity (Simple: Z = 2.640, p<.01; Complex: Z = 3.720, p<.001). In addition, Simple tasks were easier than Complex tasks when performed with the left hand (Z = 3.743, p<.001), but not when performed with the right hand (Z = 1, p = .317). Compared to unimanual conditions (derived from the study sample), Biman actions were easier than Complex-L actions (Z = 5.141, p<.001), showed a trend to be more difficult than Simple-R actions (Z = 2.659; p = .079), and were equivalent to Simple-L and Complex-R (both p>.15). Therefore, the bimanual actions were rated to be in the same difficulty range as the unimanual actions.

To test for potential movements during motor imagery, participants held two force sensitive grips in their hands [21]. Hand grip data recorded during the scanning session were averaged for each condition and participant. Force levels during the different MI conditions differed not significantly from the force levels during the baseline, with the only exception being during the Complex-L condition. In this condition, participants exerted 0.0588 N more force on the right grip than during Baseline (t(16) = 2.403, p = .029). We observed no significant differences between MI conditions, neither for the left hand, the right hand, nor the average of both hands (Fig. 1, white bars; all t(16)<1.795; all p>.05).

Directly after scanning, we assessed the subjective quality of imagination (QoI) for each movement individually on a scale from 1 (“bad/hard to imagine”) to 7 (“perfect/very vivid & lively imagination”) and calculated the median values for each condition and participant (Fig 1, gray bars). To control for the potentially confounding effect of QoI, we compared the QoI ratings of Biman with the unimanual conditions. Non-parametric Wilcoxon signed ranks tests showed that, most importantly, QoI did not differ between the bimanual and the combined unimanual conditions (Z = .877; p = .531). Comparisons of Biman with each of the four unimanual conditions further showed that QoI for Biman was significantly better than for Complex-L (Z = 2.801; p<.01), but equivalent to the remaining three unimanual conditions (all Z<1.667; all p>.180). Taken together, the quality of imagination was comparable for the bimanual and unimanual conditions.

### Univariate Approach – Localizing Increased Neural Processing

In a first step we identified brain areas generally involved in motor imagery by comparing all Imagery conditions with Baseline (i.e. ((Complex-L + Complex-R + Simple-L + Simple-R + Biman)/5) – Baseline). Because the main purpose of this contrast was to identify cortical areas for the subsequent analysis of functional connectivity, we utilized a sensitive contrast which may even reveal brain areas showing only subthreshold activation in some of the five motor imagery conditions. Consequently, areas identified by this contrast are not necessarily significantly active in all five motor imagery conditions, as would for instance be indicated by a conjunction analysis.

**Figure 2:**
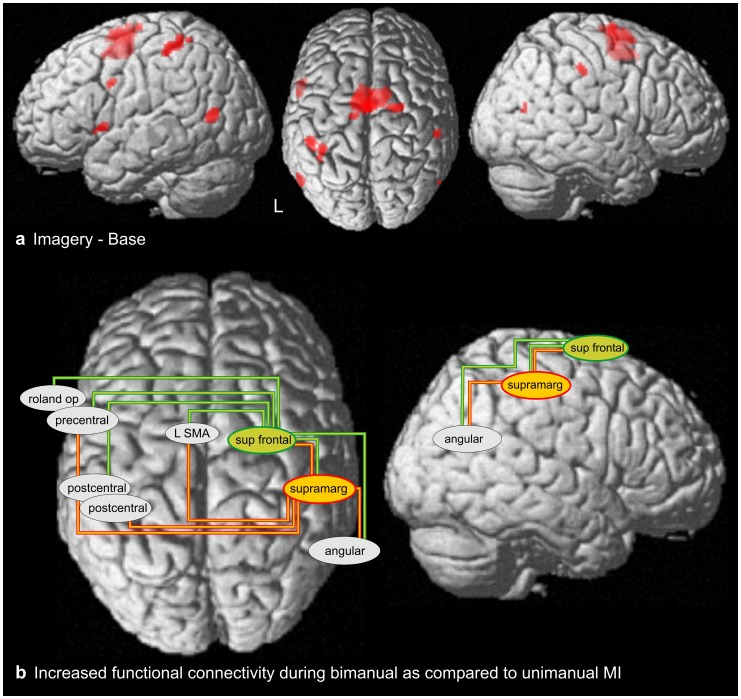
fMRI results. (**A**) Cortical areas more strongly activated during MI (averaged across all five MI conditions) than during the resting baseline (p(FWE)<.05; T(16)>7.59). Activation peaks of this contrast served as seed regions for the connectivity analysis depicted in panel B. (**B**) Increased functional connectivity during bimanual MI as compared to unimanual MI. Two seed regions exhibited increased connectivity, the right supramarginal gyrus (red) and the right superior frontal gyrus (green).

The comparison of all Imagery conditions with Baseline revealed a network of activations primarily comprising premotor and parietal areas (Table 1, Fig. 2a). In detail, premotor activation was located in the bilateral supplementary motor area (SMA; BA 6) extending into dorsal premotor cortices of both hemispheres (BA 6), and in the left rolandic operculum extending into the precentral gyrus (BA 6). Parietal activations were evident in the left postcentral gyrus, (BA 1/2), right supramarginal gyrus (BA 40) and bilateral angular (BA39) gyri. These activation peaks (Table 1) served as seed regions for the connectivity analysis (see below).

**Table 1:** Anatomical locations and MNI coordinates of activation peaks for the comparison Imagery – Baseline.

			MNI coordinate				
Location	BA	Prob	x	y	z	T	p(FWE)
L rolandic operculum	N/A	N/A	−56	10	0	9.08	0.008
L precentral G	6	70%	−58	4	32	7.92	0.033
R SMA	6	70%	2	0	52	10.07	0.002
L SMA	6	90%	−4	−8	64	13.73	0.000
R superior frontal G	6	60%	22	−8	70	10.95	0.001
R supramarg G (area PFt)	40	60%	54	−30	44	8.91	0.010
L postcentral G	1	60%	−48	−34	56	8.53	0.016
L postcentral G	2	40%	−38	−42	58	8.35	0.020
L angular G (area PGp)	N/A	20%	−58	−66	12	11.1	0.001
R angular G (area PGp)	39	50%	56	−68	18	7.99	0.030

Probability (Prob) of the location according to the Anatomy toolbox. N/A if region is not assigned by Anatomy toolbox.

Abbreviations. G  =  gyrus; SMA  =  supplementary motor area; supramarg  =  supramarginal; Prob  =  probability; BA  =  Brodmann's area; R/L  =  right/left hemispheric activation, respectively.

To test for changes in the BOLD response specific to bimanual MI, we determined over- and underadditive effects by comparing bimanual MI with the summed effects of unimanual MI [(Biman – Uni-R) – (Uni-L – Baseline)] (with Uni-R  =  (Simple-R + Complex-R)/2 and Uni-L  =  (Simple-L + Complex-L)/2). Due to the nature of this interaction contrast, which pools activity of MI of bimanual actions and the resting baseline (see section *4.5 Statistics* in the Experimental Procedures for details), we expected strong visual cortex activation, since participants had their eyes opened only during the resting baseline. The analysis revealed that except for the visual cortex, no voxels were activated with the chosen threshold of p<.05 (FWE corrected). Lowering the threshold to a more liberal criterion of p<.001 (uncorrected) with an extent threshold of 20 voxels revealed activation clusters in the cerebellar vermis, the right dorsal prefrontal cortex, and the white matter near the left hippocampus or thalamus.

Underadditive activation was evident in two areas, the left SMA (−6*x*, −6*y*, 64*z*; t(16) = 8.85; p_FWE_<.01; cluster of 53 voxel) which was assigned to BA 6 (probability 70%) by the Anatomy toolbox, and the left inferior frontal gyrus (−54*x*, 12*y*, 0*z*; t(16) = 7.87; p_FWE_<.05; 12 voxel).

Please note that part of the data (regarding laterality effects in MI, i.e. comparison of MI with the right versus the left hand) was published before [22].

### Functional Connectivity (PPI)

The PPI analysis employed the activation peaks determined in the Imagery – Baseline contrast (Table 1) as seed regions, with connectivity changes being calculated between all pairs of seed regions. This analysis showed that the contribution of two seed regions to the signal of a number of other seed regions was significantly increased under bimanual MI as compared to unimanual MI (Table 2 and Fig. 2b; there were no significant decreases in connectivity). The first seed region was located in the right supramarginal gyrus and showed increased connectivity with five regions: (1) the right superior frontal gyrus, i.e., an ipsilateral premotor area, (2) the right angular gyrus, (3) the left SMA and (4) the left precentral gyrus, i.e. two contralateral premotor regions, and (5) the left postcentral gyrus (BA 2). The second seed region was located in the right superior frontal gyrus and showed increased connectivity with six regions: (1) the right supramarginal gyrus, (2) the left postcentral gyrus (BA 1), (3) the right angular gyrus, and three contralateral (i.e., left hemispheric) premotor areas, (4) SMA, (5) precentral gyrus, and (6) rolandic operculum. This pattern suggests that inter- and intrahemispheric connectivity between parietal and premotor areas is increased when bimanual movements are imagined.

**Table 2:** Pattern of connectivity changes between seed regions (leftmost column) and target regions (top row).

	L roland operculum	L precentral G	R SMA	L SMA	R superior frontal G	R supramargin (PFt)	L postcentral G (1)	L postcentral G (2)	L angular G (PGp)	R angular G (PGp)
L rolandic operculum										
L precentral G										
R SMA										
L SMA										
R superior frontal G	L **B**	**B**	L	**B**		**B**	**B**			**B**
R supramargin (PFt)	L R	**B**		**B**	**B**			**B**		**B**
L postcentral G (1)	L R									
L postcentral G (2)			L R						R	
L angular G (PGp)	L R	L R	L				L			
R angular G (PGp)	L R	L R	L R	L R			L R		L R	

Significant changes (p<.05, FWE) for the comparisons of Uni-L vs Baseline (L), Uni-R vs Baseline (R), and Biman vs Uniman (B).

Abbreviations. G  =  gyrus; SMA  =  supplementary motor area; supramarg  =  supramarginal; Prob  =  probability; BA  =  Brodmann's area; R/L  =  right/left hemispheric activation, respectively.

In addition, we tested for changes in functional connectivity for unimanual MI by calculating PPI analyses for Uni-L vs Baseline and Uni-R vs Baseline. Like above, in both analyses connectivity changes were calculated between all pairs of seed regions identified in the Imagery – Baseline contrast (Table 1). Results (Table 2) showed that while the pattern of connectivity changes was virtually identical for both unimanual conditions Uni-L and Uni-R, it was clearly distinct from the pattern observed for Biman. Specifically, we found that in Biman the right superior frontal gyrus und right supramarginal gyrus were two seed regions changing their connectivity with a number of areas, in Uniman mainly the left and right angular gyri were such seed regions with a number of connectivity changes.

## Discussion

We investigated the neural mechanisms of MI of bimanual everyday actions. A univariate analysis revealed no evidence for significantly increased neural activity during MI of bimanual actions as compared to MI of unimanual actions. Psychophysiological interaction analyses, however, revealed a profound increase in intra- and interhemispheric functional connectivity between parietal and premotor cortices for bimanual actions. As such the data confirm our hypothesis that the increased complexity in the imagery of bimanual actions is reflected in increased connectivity between areas rather than an overall increase in neural activation.

### Cortical Areas Specifically Involved in MI of Bimanual Actions

Controlling bimanual movements places greater demands on the motor system, for instance due to the need to coordinate both limbs [12], [13]. However, the present study found no evidence for activations specific to bimanual MI. Even at a lower threshold of p<.001, uncorrected, circumscribed activations were found only in the cerebellar vermis, the white matter around the hippocampus, and the dorsolateral prefrontal cortex. Since there should be no task related fMRI signal variations in the white matter, we propose this to be an artifact. While the remaining two activations have been reported in studies investigating overt bimanual tasks [23]–[25], important components of the motor network, such as the SMA, lateral premotor cortices, or parietal cortices [12], showed no overadditive activation even when uncorrected thresholds were applied. We therefore conclude that our data provides no evidence for stronger activation of the cortical motor system specific to bimanual MI, a conclusion echoing findings on overt performance of trained bimanual actions [16].

### Cortical Areas Involved in MI

To identify seed regions for the PPI analysis, we compared all MI conditions to the baseline. The resulting network was largely in line with previous studies. For instance, the most prominent activation, i.e. bilateral SMA extending into both dorsal premotor cortices, is virtually always observed in MI [11], [26]–[30]. This finding further confirms the simulation hypothesis of MI [3], which proposes high levels of equivalence between MI and ME. Such equivalence leads to the prediction that MI should heavily rely on areas primarily associated with motor planning and movement preparation, such as the presently observed premotor areas.

The inferior parietal areas observed in the present study, i.e. the bilateral angular gyrus and the right supramarginal gyrus, have been frequently observed in MI [31]–[37]. The inferior parietal cortex mainly consists of multi-modal association areas involved in the implementation of complex actions and tool use. Damage to these areas typically results in different forms of apraxia [38]. Therefore, we think that the use of highly complex actions for MI, often involving some form of object manipulation, may have driven the inferior parietal activity in the present study.

Activation in the left postcentral gyrus may be related to the internal simulation of the tactile and haptic aspects of the imagined actions, since it has been shown that motor imagery as well as action observation can activate somatosensory cortices [39]–[41]. While the exact function of the rolandic operculum for MI is unclear, it has been observed during MI of hand movements before [28].

Interestingly, most of the brain regions listed above have been implicated in overt bimanual tasks as well. For instance, Puttemans et al. [16] identified, among other areas, the SMA, dorsal premotor cortex, rolandic operculum, and the postcentral and supramarginal gyri as associated with bimanual task performance or bimanual task learning [12].

Taken together, the activation of predominantly premotor and parietal areas is consistent with the assumed functionality of these areas for overt movement, including bimanual movements. In addition, these areas form part of the human mirror neuron system [39] and have frequently been reported in various types of MI. Thus, the present findings confirm that MI of everyday movements relies on a comparable network of brain areas as MI and overt movement of more simple laboratory tasks [17]. This finding is important, because the presently identified areas served as basis for the connectivity analysis presented next.

### Functional Connectivity of Bimanual MI

The present study is the first to test for the neural correlates of bimanual coordination demands in MI. While there was no bimanual-specific activation of motor areas, the data revealed that MI of bimanual actions increased inter- and intrahemispheric functional connectivity. In particular, the right supramarginal gyrus showed greater connectivity with the (ipsilateral) right superior frontal and angular gyrus, and with the (contralateral) left SMA and left post- and precentral gyrus. In addition, the right superior frontal gyrus showed increased connectivity with the (ipsilateral) right supramarginal and angular gyrus, and with the (contralateral) left SMA, post- and precentral gyrus, and left rolandic operculum. Taken together, all three seed regions in the right hemisphere increased their connectivity between each other. In addition, two of the seed regions in the right hemisphere (superior frontal gyrus and supramarginal gyrus) increased the connectivity to virtually all seed regions of the contralateral hemisphere. The seed regions in the left hemisphere, however, showed no increases in connectivity among each other.

We believe that this pattern is best explained by two rather independent effects, increased interhemispheric information exchange and left-hand proficiency, which are discussed in detail below.

#### Interhemispheric information exchange

The increased connectivity between the hemispheres most likely reflects the demands of coordinating the action of both hands. The everyday actions we used required not only simultaneous use of both hands, but also fine-grained interactions between the hands. A prototypical example of this is tying shoelaces [12] in which each single movement of a hand does not make sense without the concurrent movement of the other hand. This strong interdependency of the movements of the hands requires communication between the motor systems controlling each hand [13], [42], [43]. We propose that the increase in interhemispheric connectivity reflects this increased information exchange [24], [44], [45]. In more detail, for bimanual actions connectivity increased between the right supramarginal gyrus and the left pre- and postcentral gyrus and the left SMA. Complex actions are often realized by an interplay of parietal and premotor areas in one hemisphere [46]. Our findings show that this interplay becomes interhemispheric in the case of bimanual actions. The most likely interpretation of this finding is that left hemispheric premotor areas employ information represented in the right supramarginal gyrus to plan the right hand movement. In addition, the right supramarginal gyrus showed increased connectivity with the left postcentral gyrus. In which way the somatosensory information of the left hemisphere and the motor planning information of the right hemisphere interact is unclear. It might be conceivable, however, that the motor planning processes in the right supramarginal gyrus, which require positional information of the right hand [13], influence somatosensory processing in the left postcentral gyrus. Absence of the reverse pattern, i.e. left supramarginal gyrus connected with right somatosensory cortex, may be due to the fact that all our participants were right handed and that left hand imagery was less vivid (lower QoI score), which may have resulted in insufficient activity in the right somatosensory cortex [47], [48].

As the second seed region, the right superior frontal gyrus showed increased connectivity with contralateral areas, i.e. the left SMA, the left rolandic operculum, and the left pre- and postcentral gyrus. This pattern is virtually identical to the right supramarginal gyrus, with the exception that the rolandic operculum is also involved. Accordingly, these results lend further support to our hypothesis that complex actions rely on an interplay of ipsilateral premotor and parietal areas, and that this interplay spans both hemispheres in the case of bimanual actions.

#### Left-hand proficiency

The second, in our view rather independent, effect is the increase of connectivity within the right hemisphere, i.e. between right superior frontal gyrus, right supramarginal gyrus, and right angular gyrus. One reason for this finding may be that the proficiency of left hand movement in the context of left hand actions and bimanual actions is different in right-handed participants [49]. In our experiment unimanual left hand imagery comprised tasks that are typically performed with the dominant (right) hand (e.g. writing) and therefore required the imagery of relatively unfamiliar movements. The bimanual actions, however, were actions most participants did frequently and the left-hand element in these tasks was well practiced (e.g. tying shoelaces). We therefore speculate that the higher proficiency of the left hand component in bimanual movements resulted in a more vivid imagery and hence a better internal simulation of the motor act [47], [48]. The latter may explain why the cortical areas coordinating left hand action show increased connectivity in the bimanual condition [49].

An alternative explanation for the increased connectivity in the right hemisphere might be found in studies indicating that in particular the non-dominant hemisphere (i.e., the right-hemisphere in the present study) is involved in the overt execution of bimanual movements [50], [51]. Interestingly, this suggestion has been derived based on the effects of lesions [50] or disruptive TMS [51] on bimanual motor performance and is therefore compatible with our argument that the demands of bimanual coordination may be reflected mainly by increased connectivity rather than distinct activation patterns.

### Previous Evidence on Connectivity of Overt Bimanual Movements

As there are no studies on the connectivity of bimanual MI, we discuss studies using overt bimanual movements. Sun et al., (2007) compared a bimanual task to a resting baseline and observed a network only partially overlapping with the presently observed activations, consisting of primary sensorimotor cortices, dorsal premotor cortices, dorsal prefrontal cortices, intraparietal sulci, SMA, cingulate motor area, and cuneus. When compared to a resting baseline, the bimanual condition resulted in increased connectivity between a number of areas. In particular, the dorsal premotor cortex showed increased connectivity with the respective other, contralateral dorsal premotor cortex, the ipsilateral SMA, bilateral sensorimotor cortices, and the posterior parietal cortex. Further seed regions in the sensorimotor cortex, the SMA, and the intraparietal sulcus also showed increased inter- and intrahemispheric connectivity. Thus, while there are differences in the exact components involved in the cortical network controlling bimanual actions, Sun and colleagues also observed increased connectivity between premotor and (superior) parietal areas within and between hemispheres.

There are a number of factors that could explain the differences in findings between our and Sun et al’s study [24], most notably the fact that we used MI while Sun et al. used overt movements. Overt and imagined movements rely on an overlapping network, however, clear differences have been noted as well [34], [52]. Moreover, in Sun et al.’s study the bimanual condition was compared to a rest condition and to another bimanual condition. For the comparison with rest, it cannot be ruled out that the same changes in connectivity would have been observed with a unimanual task. Therefore, our results, which were derived by comparison with unimanual conditions, extend the previous knowledge by showing changes in connectivity unequivocally associated with bimanual MI. Finally, the task employed by Sun et al. required no simultaneous bimanual action, but a pattern of alternating button presses (one hand after the other). While the effect of this difference in tasks on the pattern of connectivity is presently unclear, it is interesting to note that the exact amount of temporal overlap of two actions can profoundly influence the processing demands associated with a task [53]–[55].

A further study investigating connectivity in overt bimanual movements was conducted by Grefkes et al. [14]. In this study, participants had to make fist movements with the left, right, or both hands, and connectivity changes were assessed between 6 ROIs (SMA, premotor cortex, and primary motor cortex of each hemisphere, respectively) using DCM. First, it is interesting to note that Grefkes et al. also found only weak evidence for increased neural processing during bimanual as compared to unimanual performance. A comparison of bimanual with unimanual tasks revealed, however, profound changes in functional connectivity. In particular, connectivity increased intra- and interhemispherically between the SMA and primary motor cortex, while the premotor cortex showed only intrahemispheric increases with SMA and primary motor cortex. The parallel between the two studies is the finding of increased connectivity between the right premotor cortex and the left SMA. While the premotor cortices did not show interhemispheric connectivity changes in Grefkes et al., they did so in the present study. This discrepancy may be due to the different modalities of motor stimulation (imagery vs execution). Alternatively, the bimanual movements employed by us (e.g. tying shoelaces) required much stronger interlimb coordination than the movements used by Grefkes et al., i.e. fist making, [12], which may have resulted in increased demands on interhemispheric coordination. On a broader level, however, the results of Grefkes et al. and the present study converge in that both show that the demands of bimanual coordination are met by inter- and intrahemispheric changes in connectivity and not by changes in activation level.

### Clinical Aspects

The current findings are relevant for the rehabilitation of patients suffering from motor deficits in at least three aspects. First, some patients’ residual movement abilities are too poor to permit standard rehabilitation. For such patients, MI may be a way to initiate recovery, at least to a level at which standard approaches with overt movement are possible [56]–[59]. However, for MI to be most effective a vivid mental image is required, which may be easier to generate when everyday actions are used which have been performed numerous times before the brain damage occurred [60], [61]. For instance, Fourkas et al. [18] demonstrated that during kinesthetic motor imagery corticospinal facilitation was present only in experts imagining the movement of their expertise. Critically, the present results show that MI of everyday actions not only engages the premotor system in terms of activation, but highlight that bimanual MI also increases the functional connectivity between premotor areas.

This finding of increased connectivity in bimanual MI is perfectly in line with the observation that bimanual rehabilitation procedures can be more beneficial for recovery than unimanual procedures [62]–[64], presumably because the affected motor system is facilitated (or disinhibited) by the intact motor system [65], [66]. At the same time, recent evidence suggests a positive association between connectivity and recovery [14], [67]. Although it is presently unclear whether loss of connectivity limits motor recovery or, alternatively, improving motor recovery increases connectivity, it seems promising, given the present results, to develop a training regime based on MI which explicitly aims for improving the connectivity between areas, e.g. based on bimanual training.

Our finding of additional increases of connectivity beyond the ones observed during unimanual MI might further suggest that.bimanual MI is a more effective form of covert movement for rehabilitation. In more detail, the performance of unimanual MI resulted in increased connectivity between several areas involved in MI. Thereby the connectivity changes during bimanual MI were quite distinct from those observed for unimanual MI, i.e. they were between different pairs of seed regions. This dissociation is in line with our suggestion that bimanual coordination requires additional processes not demanded during unimanual MI. It is an intriguing question for future research whether this additional connectivity causally improves motor rehabilitation.

The present results were based on investigating healthy participants and should be confirmed in neurological patients [68]. In addition, one should be aware that certain brain areas, such as specific parts of the parietal cortex, need to be intact to ensure that patients can properly perform MI [37], [69]. However, these rather minor limitations should not disregard the clinical potential of using bimanual MI in motor rehabilitation.

## Materials and Methods

### Ethics Statement

Prior to scanning, written informed consent was obtained. The protocol was approved by the University of Surrey ethical review board.

### Participants

17 neurologically healthy participants (6 male), aged 19 to 31 years (mean 22), took part in the experiment. All participants were right handed with a mean handedness score of 82, range 53–100, as assessed with the Edinburgh Inventory [70]. Participants received £ 15 for participation.

### Task and Procedure

While lying in the MRI-scanner, participants viewed a projection screen via a mirror attached to the head coil with a distance of approximately 2–5 cm to the eyes. The display was back-projected onto a 60cm-diameter screen situated approximately 30–40 cm away from the mirror.

**Figure 3:**
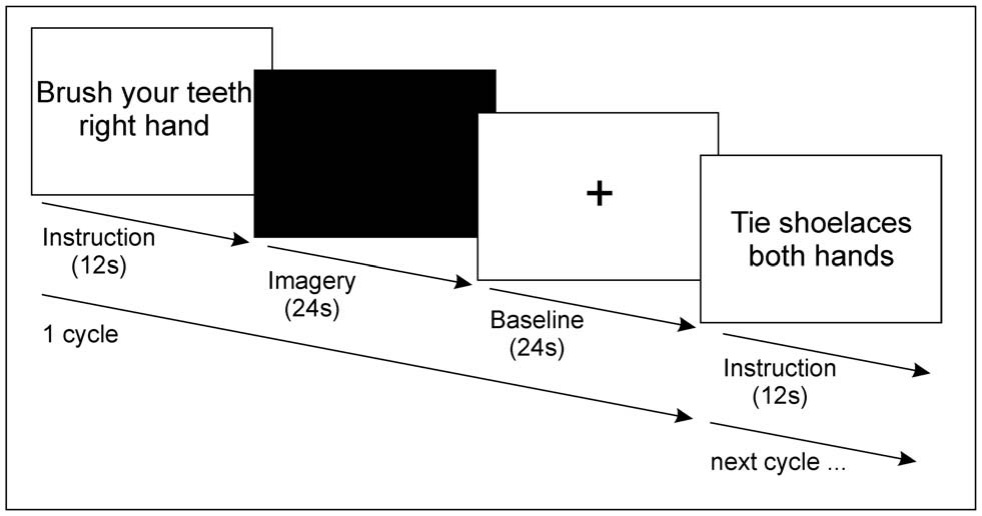
Trial design.

The paradigm comprised five conditions, Biman, Simple-L, Simple-R, Complex-L, Complex-R, and Baseline. Except for Baseline, each condition was repeated seven times. The experiment used a block design consisting of 35 one-minute cycles. Each cycle embodied an instruction and preparation period (12 s), an imagination period (24 s), and a resting baseline period (Baseline, 24 s) (Figure 3). To optimize BOLD signal recovery the unimanual imagery conditions were presented in two basic patterns alternating hand and complexity, i.e.: Simple-L, Complex-R, Simple-L, Complex-R, and so forth and Simple-R, Complex-L, Simple-R, Complex-L, and so forth. The randomization pattern was switched after every Biman condition, which was presented randomly every third to fifth cycle. The experiment was split in two runs of 18 min (18 cycles) and 17 min (17 cycles) respectively.

In the instruction period the movement to be imagined next was presented on a screen using black letters on white background. Participants were instructed to use this period to prepare the imagination by setting up an action plan. Commencement of the imagination period was indicated by the screen turning black. Participants were asked to close their eyes to perform the imagination. Participants were instructed to open the eyes again when the screen turned white, which, due to the intense change in luminance, was easy to recognize through the closed eye lids. The imagination period was followed by a baseline period (Baseline), during which participants had to fixate a cross on the screen. After this baseline period, the next cycle started with the instruction and preparation period.

To test for potential movements during motor imagery, participants held two custom made force sensitive grips in their hands [21] which acquired data continuously throughout all conditions with a 250 Hz sampling rate. The grips are highly sensitive to force changes and are able to detect force variations not visible by visual inspection. Typically, participants held the grip so that thumb and fingers were opposed and force variations were identified for all five fingers. However, during the session some participants may have changed the way they held the grip so that force variations of the thumb may have stayed undetected. While it could be argued that holding the grips is in itself a motor act and, therefore, may interfere with the vivid generation of kinesthetic MI, we think that this is unlikely to affect our results for two reasons. Firstly, participants held the grip loosely, resting their hands on the scanner bed and gently closing their hands around the grip. Secondly, it seems plausible to assume that MI performance would be affected in all MI conditions. We therefore argue that any bias induced by holding the grips would be constant across conditions and hence would not confound differential effects between conditions.

Participants were instructed to imagine the movements in a kinesthetic first person perspective, i.e. they were asked to imagine performing the movement by themselves, rather than watching themselves or others performing the movement [6], [71]. The instruction further emphasized that the imagination should be “action loaded”, i.e. participants should perform the imagined movement with high frequency and engage intensely. Participants were instructed to actively imagine throughout the imagination period and, if a movement finished early, to start over with the same movement until the imagination period finished.

The movements of the Biman condition were (1) Tie shoelaces, (2) Button a shirt or blouse, (3) Fold a letter and put in an envelope, (4) Fold laundry, (5) Tear paper apart, (6) Pull up socks, (7) Dry your back using a towel. The simple unimanual movements (Simple-L and -R) were (1) Scratch your nose (2) Use a light switch (turn light on and off) (3) Open cupboard door (left/right door if performed with left/right hand, respectively), (4) Drink glass of water placed on a table, (5) Press a button (e.g. in a lift), (6) Turn round knob (e.g. volume control at HiFi), (7) Hang your coat on a hook. The complex unimanual movements (Complex-L and -R) were (1) Write on a piece of paper using a pen, (2) Brush your teeth, (3) Use a computer mouse, (4) Eat soup or cereals using a spoon, (5) Throw something (in the trash bin/darts), (6) Lock/unlock a door using a key, (7) Shake hands.

To assess the difficulty of the movements, participants rated the perceived difficulty for each movement when performed with the left and right hand, respectively, using a scale ranging from 1 (“very easy”) to 5 (“very hard”). This rating took place before the experiment and asked the participants to rate how difficult it would be to overtly perform the respective movements. We tested for significant differences between conditions using non-parametric Wilcoxon tests. Retrospectively an independent sample of 17 participants completed an adapted version of this questionnaire which included items relating to difficulty of bimanual actions as we did not test this particular aspect prior to the study. There were no significant differences between the original study group and the independent set of participants regarding the estimated difficulty of the four unimanual conditions (Mann-Whitney U test; all Z<1.560; all p>.193). Accordingly, we treated the estimated difficulty of the Biman condition derived from the independent sample as representative for the study sample. All comparisons between bimanual and unimanual conditions employed the independent-samples Mann-Whitney U test.

Vividness of the imagery was assessed through a short questionnaire completed immediately after the MRI scanning, which measured the subjective quality of the imagination (QoI) during the experiment on a scale from 1 (“bad/hard to imagine”) to 7 (“perfect/very vivid & lively imagination”).

### MRI Procedure

Imaging was carried out at the Royal Holloway University London, UK, using a 3T scanner (Trio, Siemens, Erlangen, Germany) equipped with an array head coil. Participants were supine on the scanner bed, and cushions were used to reduce head motion. 36 axial slices (192×192 mm field of view (FOV), 64×64 matrix, 3×3 mm in-plane resolution, 4 mm thickness, no gap, interleaved slice acquisition) were acquired using a BOLD sensitive gradient echo EPI sequence (TR 2 s, TE 30 ms, 90° flip angle). Two functional runs, the first with 540 and the second with 510 volumes were administered, with each volume sampling all 36 slices. In the same session, high-resolution whole brain images were acquired using a T1-weighted MPRAGE sequence (TR 1830 ms, TE 4.43 ms, 11° flip angle, 176 slices, 256×256 mm FOV, 1×1×1 mm voxel size).

### Data Analysis

#### Preprocessing

The data were analyzed using the SPM2 software package (http://www.fil.ion.ucl.ac.uk/spm/software/spm2/). In a first step, the origin of the functional images was manually set to the anterior commissure and all images were reoriented. To correct for movements, all functional volumes were spatially realigned to the first functional volume. In the same processing step (“Realign & Unwarp” in SPM2), signal changes due to head motion and magnetic field inhomogenities were corrected [72]. Next, the normalization was performed. For this, first the anatomical and functional images were co-registered, then the anatomical image was normalized into a standard stereotaxic space using the T1 template provided by the Montreal Neurological Institute (MNI) delivered with SPM, and finally the transformation parameters derived from this transformation were applied to the functional images. Functional data were spatially smoothed using a Gaussian kernel with a FWHM of 8 mm.

### Statistics

#### Univariate approach

Statistical analysis was based on a voxelwise least squares estimation using the general linear model for serially autocorrelated observations [19], [73]. All conditions (including Baseline) were modeled using the standard hemodynamic response function implemented in SPM2. Low-frequency signal drifts were controlled for by applying a temporal highpass filter with a cutoff frequency of 1/300 Hz. To test for imagery related activation individual contrast maps were calculated for the comparison Imagery – Baseline (i.e. ((Complex-L + Complex-R + Simple-L + Simple-R + Biman)/5) – Baseline). To test for activations specific to MI of bimanual actions we considered our design as a 2×2 factorial design with the factors MI of right hand (levels present/absent) and MI of left hand (levels present/absent) [15]. Both hands absent reflects the resting baseline (Baseline), only left or right hand present reflect the two unimanual conditions, and both hands present reflects the Biman condition. This design enables to test for overadditive activation in Biman which cannot be reduced to the summed activations of the two unimanual conditions by the interaction contrast [(Biman – Uni-R) – (Uni-L – Baseline)], with Uni-R  =  (Simple-R + Complex-R)/2 and Uni-L  =  (Simple-L + Complex-L)/2. Resolving the brackets results in the comparison (Biman + Baseline – Uni-R – Uni-L). The second-level analysis consisted of random-effects paired t-tests with p value threshold set to p<.05 (FWE corrected for multiple comparisons). Anatomical locations were determined using the Anatomy toolbox version 1.6 [74].

#### Multivariate connectivity analysis

A multi psychophysiologic interaction (mPPI) protocol was implemented using the process of signal deconvolution embedded in SPM2 [20]. Firstly, we identified seed regions of interest based on the peak activations of clusters derived from the second-level contrast Imagery – Baseline of the univariate approach (*p*<0.05, FWE). For each subject, the largest effect of this contrast (individual SPMs generated at *p*<0.001, uncorrected) was found within a four millimeter radius of the coordinate derived from the second level effect, and this new coordinate became the individual's seed for that region. The first eigenvariables were calculated for each individual seed (sphere with 4mm radius to maintain signal specificity, Gonçalves & Hall, 2003) and constituted the physiological component. The psychological component was modeled as a Biman > Uniman (averaged across Simple and Complex unimanual tasks) contrast. The design matrix composed four regressors per session. The interaction between the psychological component and the physiological component was used as the regressor of interest. Additionally, both the psychological and physiological components were input as regressors of no interest. The fourth regressor constituted the error term. The contrast of the effect of the PPI was calculated for each subject. Each of the subject-specific contrasts, for each ROI, was subjected to a second level analysis in which one-sample t tests were calculated. Therefore, each seed region yielded one second level set of results. A mask image was created which included all voxels within 8mm of a seed voxel. Statistical Parametric Maps (SPMs) were calculated within these mask regions and corrected accordingly using the SPM2 small volume correction tool (p<.05, FWE).

## References

[1] Crammond DJ (1997). Motor imagery: never in your wildest dream.. Trends Neurosci.

[2] Decety J (1996). Do imagined and executed actions share the same neural substrate?. Brain Res Cogn Brain Res.

[3] Jeannerod M (1994). The representing brain: neural correlates of motor intention and imagery.. Behavioral and Brain Sciences.

[4] Decety J, Jeannerod M (1995). Mentally simulated movements in virtual reality: does Fitts's law hold in motor imagery?. Behav Brain Res.

[5] Pascual-Leone A, Nguyet D, Cohen LG, Brasil-Neto JP, Cammarota A (1995). Modulation of muscle responses evoked by transcranial magnetic stimulation during the acquisition of new fine motor skills.. J Neurophysiol.

[6] Stinear CM, Byblow WD, Steyvers M, Levin O, Swinnen SP (2006). Kinesthetic, but not visual, motor imagery modulates corticomotor excitability.. Exp Brain Res.

[7] Miller KJ, Schalk G, Fetz EE, den Nijs M, Ojemann JG (2010). Cortical activity during motor execution, motor imagery, and imagery-based online feedback.. Proc Natl Acad Sci U S A.

[8] Kranczioch C, Mathews S, Dean PJ, Sterr A (2009). On the equivalence of executed and imagined movements: evidence from lateralized motor and nonmotor potentials.. Hum Brain Mapp.

[9] Lotze M, Halsband U (2006). Motor imagery.. J Physiol Paris.

[10] Pfurtscheller G, Neuper C (1997). Motor imagery activates primary sensorimotor area in humans.. Neurosci Lett.

[11] Porro CA, Francescato MP, Cettolo V, Diamond ME, Baraldi P (1996). Primary motor and sensory cortex activation during motor performance and motor imagery: A functional magnetic resonance imaging study.. Journal of Neuroscience.

[12] Swinnen SP (2002). Intermanual coordination: from behavioural principles to neural-network interactions.. Nat Rev Neurosci.

[13] Guiard Y (1987). Asymmetric division of labor in human skilled bimanual action: the kinematic chain as a model.. J Mot Behav.

[14] Grefkes C, Eickhoff SB, Nowak DA, Dafotakis M, Fink GR (2008). Dynamic intra- and interhemispheric interactions during unilateral and bilateral hand movements assessed with fMRI and DCM.. Neuroimage.

[15] Szameitat AJ, Schubert T, Müller HJ (2011). How to test for dual-task-specific effects in brain imaging studies–an evaluation of potential analysis methods.. Neuroimage.

[16] Puttemans V, Wenderoth N, Swinnen SP (2005). Changes in brain activation during the acquisition of a multifrequency bimanual coordination task: from the cognitive stage to advanced levels of automaticity.. J Neurosci.

[17] Szameitat AJ, Shen S, Sterr A (2007). Motor imagery of complex everyday movements. An fMRI study.. Neuroimage.

[18] Fourkas AD, Bonavolonta V, Avenanti A, Aglioti SM (2008). Kinesthetic imagery and tool-specific modulation of corticospinal representations in expert tennis players.. Cereb Cortex.

[19] Friston KJ, Holmes AP, Worsley KJ, Poline J-P, Frith CD (1995). Statistical Parametric Maps in Functional Imaging: A General Linear Approach.. Human Brain Mapping.

[20] Friston KJ, Büchel C, Fink GR, Morris J, Rolls E (1997). Psychophysiological and modulatory interactions in neuroimaging.. NeuroImage.

[21] Hou W, Shen S, Sterr A (2005). An MRI compatible visual force-feedback system for the study of force control mechanics.. Conf Proc IEEE Eng Med Biol Soc.

[22] Szameitat AJ, Shen S, Sterr A (2007). Effector-dependent activity in the left dorsal premotor cortex in motor imagery.. Eur J Neurosci.

[23] Debaere F, Wenderoth N, Sunaert S, Van Hecke P, Swinnen SP (2004). Cerebellar and premotor function in bimanual coordination: parametric neural responses to spatiotemporal complexity and cycling frequency.. Neuroimage.

[24] Sun FT, Miller LM, Rao AA, D'Esposito M (2007). Functional connectivity of cortical networks involved in bimanual motor sequence learning.. Cereb Cortex.

[25] Wenderoth N, Toni I, Bedeleem S, Debaere F, Swinnen SP (2006). Information processing in human parieto-frontal circuits during goal-directed bimanual movements.. Neuroimage.

[26] Bakker M, De Lange FP, Helmich RC, Scheeringa R, Bloem BR (2008). Cerebral correlates of motor imagery of normal and precision gait.. Neuroimage.

[27] Hanakawa T, Immisch I, Toma K, Dimyan MA, Van Gelderen P (2003). Functional properties of brain areas associated with motor execution and imagery.. J Neurophysiol.

[28] Kuhtz-Buschbeck JP, Mahnkopf C, Holzknecht C, Siebner H, Ulmer S (2003). Effector-independent representations of simple and complex imagined finger movements: a combined fMRI and TMS study.. Eur J Neurosci.

[29] Michelon P, Vettel JM, Zacks JM (2006). Lateral somatotopic organization during imagined and prepared movements.. J Neurophysiol.

[30] Naito E, Kochiyama T, Kitada R, Nakamura S, Matsumura M (2002). Internally simulated movement sensations during motor imagery activate cortical motor areas and the cerebellum.. J Neurosci.

[31] Boecker H, Ceballos-Baumann AO, Bartenstein P, Dagher A, Forster K (2002). A H(2)(15)O positron emission tomography study on mental imagery of movement sequences–the effect of modulating sequence length and direction.. Neuroimage.

[32] Dechent P, Merboldt KD, Frahm J (2004). Is the human primary motor cortex involved in motor imagery?. Brain Res Cogn Brain Res.

[33] Deiber MP, Ibanez V, Honda M, Sadato N, Raman R (1998). Cerebral processes related to visuomotor imagery and generation of simple finger movements studied with positron emission tomography.. Neuroimage.

[34] Gerardin E, Sirigu A, Lehericy S, Poline JB, Gaymard B (2000). Partially overlapping neural networks for real and imagined hand movements.. Cereb Cortex.

[35] Stephan KM, Fink GR, Passingham RE, Silbersweig D, Ceballos-Baumann AO (1995). Functional anatomy of the mental representation of upper extremity movements in healthy subjects.. J Neurophysiol.

[36] Yoo SS, Freeman DK, McCarthy JJ, 3rd, Jolesz FA (2003). Neural substrates of tactile imagery: a functional MRI study.. Neuroreport.

[37] Fleming MK, Stinear CM, Byblow WD (2010). Bilateral parietal cortex function during motor imagery.. Exp Brain Res.

[38] Goldenberg G (2009). Apraxia and the parietal lobes.. Neuropsychologia.

[39] Gazzola V, Keysers C (2009). The observation and execution of actions share motor and somatosensory voxels in all tested subjects: single-subject analyses of unsmoothed fMRI data.. Cereb Cortex.

[40] Malouin F, Richards CL, Jackson PL, Dumas F, Doyon J (2003). Brain activations during motor imagery of locomotor-related tasks: a PET study.. Hum Brain Mapp.

[41] Wolbers T, Weiller C, Buchel C (2003). Contralateral coding of imagined body parts in the superior parietal lobe.. Cereb Cortex.

[42] Debaere F, Wenderoth N, Sunaert S, Van Hecke P, Swinnen SP (2004). Changes in brain activation during the acquisition of a new bimanual coodination task.. Neuropsychologia.

[43] Stephan KM, Binkofski F, Halsband U, Dohle C, Wunderlich G (1999). The role of ventral medial wall motor areas in bimanual co-ordination. A combined lesion and activation study.. Brain 122 (Pt.

[44] Andres FG, Mima T, Schulman AE, Dichgans J, Hallett M (1999). Functional coupling of human cortical sensorimotor areas during bimanual skill acquisition.. Brain 122 (Pt.

[45] Gerloff C, Andres FG (2002). Bimanual coordination and interhemispheric interaction.. Acta Psychol (Amst).

[46] Cabeza R, Nyberg L (2000). Imaging cognition II: An empirical review of 275 PET and fMRI studies.. Journal of Cognitive Neuroscience.

[47] Olivetti Belardinelli M, Palmiero M, Sestieri C, Nardo D, Di Matteo R (2009). An fMRI investigation on image generation in different sensory modalities: the influence of vividness.. Acta Psychol (Amst).

[48] Lorey B, Pilgramm S, Bischoff M, Stark R, Vaitl D (2011). Activation of the parieto-premotor network is associated with vivid motor imagery–a parametric FMRI study.. PLoS One.

[49] Gao Q, Duan X, Chen H (2011). Evaluation of effective connectivity of motor areas during motor imagery and execution using conditional Granger causality.. Neuroimage.

[50] Halsband U, Ito N, Tanji J, Freund HJ (1993). The role of premotor cortex and the supplementary motor area in the temporal control of movement in man.. Brain 116 (Pt.

[51] van den Berg FE, Swinnen SP, Wenderoth N (2010). Hemispheric asymmetries of the premotor cortex are task specific as revealed by disruptive TMS during bimanual versus unimanual movements.. Cereb Cortex.

[52] Macuga KL, Frey SH (2012). Neural representations involved in observed, imagined, and imitated actions are dissociable and hierarchically organized.. Neuroimage.

[53] Pashler H (1994). Dual-task interference in simple tasks: Data and theory.. Psychological Bulletin.

[54] Stelzel C, Brandt SA, Schubert T (2009). Neural mechanisms of concurrent stimulus processing in dual tasks.. Neuroimage.

[55] Szameitat AJ, Schubert T, Müller K, von Cramon DY (2002). Localization of executive functions in dual-task performance with fMRI.. J Cogn Neurosci.

[56] Ertelt D, Small S, Solodkin A, Dettmers C, McNamara A (2007). Action observation has a positive impact on rehabilitation of motor deficits after stroke.. Neuroimage.

[57] Pomeroy VM, Clark CA, Miller JS, Baron JC, Markus HS (2005). The potential for utilizing the "mirror neurone system" to enhance recovery of the severely affected upper limb early after stroke: a review and hypothesis.. Neurorehabil Neural Repair.

[58] Sharma N, Pomeroy VM, Baron JC (2006). Motor imagery: a backdoor to the motor system after stroke?. Stroke.

[59] Garrison KA, Winstein CJ, Aziz-Zadeh L (2010). The mirror neuron system: a neural substrate for methods in stroke rehabilitation.. Neurorehabil Neural Repair.

[60] Olsson CJ, Nyberg L (2010). Motor imagery: if you can't do it, you won't think it.. Scand J Med Sci Sports.

[61] Toussaint L, Blandin Y (2010). On the role of imagery modalities on motor learning.. J Sports Sci.

[62] Cauraugh JH (2004). Coupled rehabilitation protocols and neural plasticity: upper extremity improvements in chronic hemiparesis.. Restor Neurol Neurosci.

[63] Gordon AM, Schneider JA, Chinnan A, Charles JR (2007). Efficacy of a hand-arm bimanual intensive therapy (HABIT) in children with hemiplegic cerebral palsy: a randomized control trial.. Dev Med Child Neurol.

[64] Renner CI, Woldag H, Atanasova R, Hummelsheim H (2005). Change of facilitation during voluntary bilateral hand activation after stroke.. J Neurol Sci.

[65] Stinear JW, Byblow WD (2002). Disinhibition in the human motor cortex is enhanced by synchronous upper limb movements.. J Physiol.

[66] Woldag H, Lukhaup S, Renner C, Hummelsheim H (2004). Enhanced motor cortex excitability during ipsilateral voluntary hand activation in healthy subjects and stroke patients.. Stroke.

[67] Sharma N, Baron JC, Rowe JB (2009). Motor imagery after stroke: relating outcome to motor network connectivity.. Ann Neurol.

[68] Stinear CM, Fleming MK, Barber PA, Byblow WD (2007). Lateralization of motor imagery following stroke.. Clin Neurophysiol.

[69] Sirigu A, Duhamel JR, Cohen L, Pillon B, Dubois B (1996). The mental representation of hand movements after parietal cortex damage.. Science.

[70] Oldfield RC (1971). The assessment and analysis of handedness: The Edinburgh Inventory.. Neuropsychologia.

[71] Solodkin A, Hlustik P, Chen EE, Small SL (2004). Fine modulation in network activation during motor execution and motor imagery.. Cereb Cortex.

[72] Andersson JL, Hutton C, Ashburner J, Turner R, Friston K (2001). Modeling geometric deformations in EPI time series.. Neuroimage.

[73] Friston KJ, Holmes AP, Poline JB, Grasby PJ, Williams SC (1995). Analysis of fMRI time-series revisited.. NeuroImage.

[74] Eickhoff SB, Stephan KE, Mohlberg H, Grefkes C, Fink GR (2005). A new SPM toolbox for combining probabilistic cytoarchitectonic maps and functional imaging data.. Neuroimage.

